# Gonadal Sex Differentiation: Supporting Versus Steroidogenic Cell Lineage Specification in Mammals and Birds

**DOI:** 10.3389/fcell.2020.616387

**Published:** 2020-12-18

**Authors:** Martin A. Estermann, Andrew T. Major, Craig A. Smith

**Affiliations:** Department of Anatomy and Developmental Biology, Monash Biomedicine Discovery Institute, Monash University, Clayton, VIC, Australia

**Keywords:** sex determination, testis, ovary, single cell RNA seq, chicken embryo, gonad, gonadal differentiation

## Abstract

The gonads of vertebrate embryos are unique among organs because they have a developmental choice; ovary or testis formation. Given the importance of proper gonad formation for sexual development and reproduction, considerable research has been conducted over the years to elucidate the genetic and cellular mechanisms of gonad formation and sexual differentiation. While the molecular trigger for gonadal sex differentiation into ovary of testis can vary among vertebrates, from egg temperature to sex-chromosome linked master genes, the downstream molecular pathways are largely conserved. The cell biology of gonadal formation and differentiation has long thought to also be conserved. However, recent discoveries point to divergent mechanisms of gonad formation, at least among birds and mammals. In this mini-review, we provide an overview of cell lineage allocation during gonadal sex differentiation in the mouse model, focusing on the key supporting and steroidogenic cells and drawing on recent insights provided by single cell RNA-sequencing. We compare this data with emerging information in the chicken model. We highlight surprising differences in cell lineage specification between species and identify gaps in our current understanding of the cell biology underlying gonadogenesis.

## Introduction

Gonadal sex differentiation in vertebrates typically results in either ovary or testis formation. This is a significant event in embryogenesis, setting the stage for either female or male development. Gonads are initially morphologically identical between the sexes (the so-called “bipotential or “indifferent” stage) and are subsequently directed down the ovarian or testicular pathways (Nef et al., 2005; Barseghyan et al., 2018; Stevant et al., 2019). Different genetic or environmental cues can initiate these alternative pathways (Trukhina et al., 2013). Therian mammals have an XX:XY sex chromosome system and the Y chromosome-linked *SRY* gene acts as the master sex determinant, directing testis formation. However, *SRY* is absent in non-mammals. Birds have ZZ:ZW sex chromosomes and gonadal sex determination is governed by the Z-linked gene, *DMRT1*, which operates via a dosage mechanism (Smith et al., 2009; Ioannidis et al., 2020). In many reptiles, egg incubation temperature governs the direction of gonadal sex differentiation (Yao et al., 2004a; Merchant-Larios and Diaz-Hernandez, 2013; Georges and Holleley, 2018). In these species, temperature has an epigenetic effect upon the regulation of genes such as *DMRT1* (Ge et al., 2017, 2018). Teleost fish exhibit a remarkable variety of different genetic sex determining triggers, (Matsuda et al., 2002; Hattori et al., 2012; Liew et al., 2012; Crespo et al., 2013; Bertho et al., 2018).

**Figure 1:**
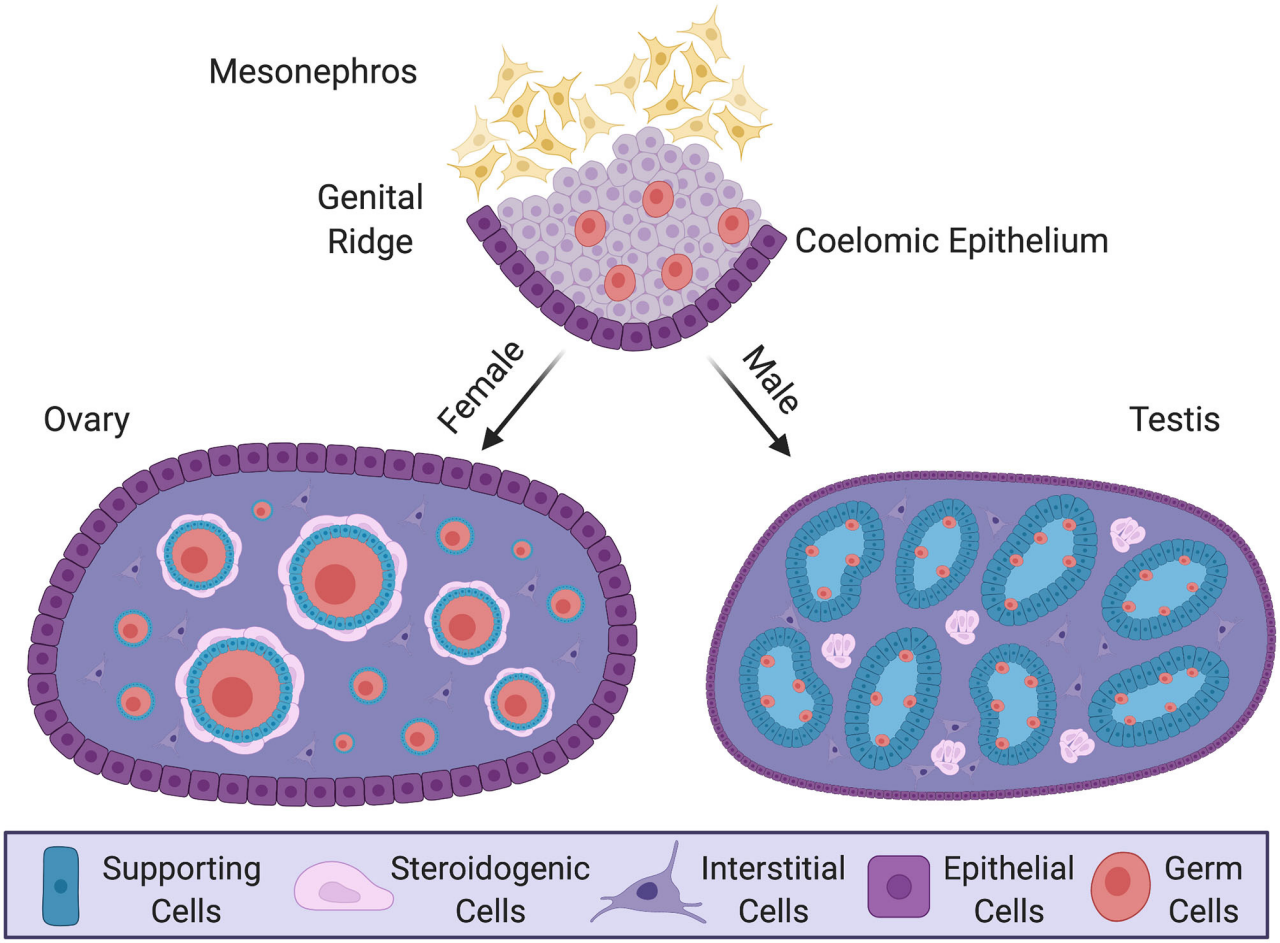
Gonadal sex differentiation and cell lineage specification in the mouse model. Development of the gonad at embryonic and postnatal stages in the mouse. In the embryo, the genital ridge forms as a thickening of coelomic epithelium overlying loose mesenchyme of the mesonephric kidney. Proliferating epithelium cells generate a pool of multipotent progenitor cells. In male (XY) postnatal gonad (testis), the progenitors give rise to pre-Sertoli cells, which surround germ cells and organize into testis cords. Steroidogenic fetal Leydig cells, together with some non-steroidogenic cells, differentiate around these cords. In the female postnatal gonad (ovary) supporting cells differentiate as granulosa cells, encircling meiotically arrested germ cells to form primordial follicles. Steroidogenic thecal cells differentiate around these structures. The supporting and steroidogenic cells in males and females are homologous, having have common origins. Key shows the different cell types.

Despite these diverse triggers for gonadal sex differentiation, most downstream genes are conserved among vertebrates. These include the transcription factor gene *SOX9* and the hormone *AMH*, up-regulated in developing testes (Kent et al., 1996; Nishikimi et al., 2000; Torres Maldonado et al., 2002), and the signaling factors *WNT4, R-SPO1*, and forkhead transcription factor *FOXL2*, up-regulated in developing ovaries (Loffler et al., 2003; Smith et al., 2008; Bertho et al., 2016; Hirst et al., 2017; Zhang et al., 2017; Major et al., 2019; Yamashita et al., 2019). Similarly, the cellular composition of the gonads is conserved. In all vertebrates, the gonadal primordium largely consists of so-called supporting cell precursors, steroidogenic progenitors, and primordial germ cells (Stevant et al., 2018; Nef et al., 2019). During gonadal sex differentiation, each gonadal cell population commits to an ovarian or testicular cell fate (Albrecht and Eicher, 2001; Chen et al., 2017). In the developing testis, the supporting cell lineage differentiates into Sertoli cells, which enclose germ cells and form testis cords (Figure 1) (Rebourcet et al., 2014). In the surrounding interstitium, the steroidogenic lineage differentiates into testosterone-producing fetal Leydig cells (Barsoum and Yao, 2011; Zhang et al., 2015; Liu et al., 2016). In the ovary, supporting cell precursors form pre-granulosa cells, steroidogenic progenitors become thecal cells and germ cells differentiate into oogonia (Mork et al., 2012; Hummitzsch et al., 2013; Liu et al., 2015; Wear et al., 2017) (Figure 1). The supporting cell lineage is the first to differentiate under the direction of a sex-determining trigger, such as *Sry* (Albrecht and Eicher, 2001; Wilhelm et al., 2005). This lineage is thought to channel other cell lineages down the ovarian or testicular pathway. However, recent studies are shedding new light on cell lineage allocation during vertebrate gonadal sex differentiation. These studies are showing that gonadal development is more complex than previously thought, involving more cell types and, surprisingly, that their derivation may differ among different vertebrate lineages (Stevant et al., 2018; Estermann et al., 2020; Niu and Spradling, 2020). In this mini-review, we summarize recent developments in this area, focusing on the somatic component of the gonad and, in particular, the application of single cell transcriptomics for tracing the origin of gonadal cell types and delineating cell fate trajectories (Stevant et al., 2019). We identify gaps in existing knowledge and outline current directions. As vertebrate gonadal sex (testis vs. ovary) is typically determined by somatic cells, not the germ cells, we focus here on this component. However, it is noted that germ cells play an essential role in gonadal sex determination in some models, such as zebrafish (Slanchev et al., 2005; Siegfried and Nusslein-Volhard, 2008).

## The Undifferentiated Gonadal Primordium

**Figure 2:**
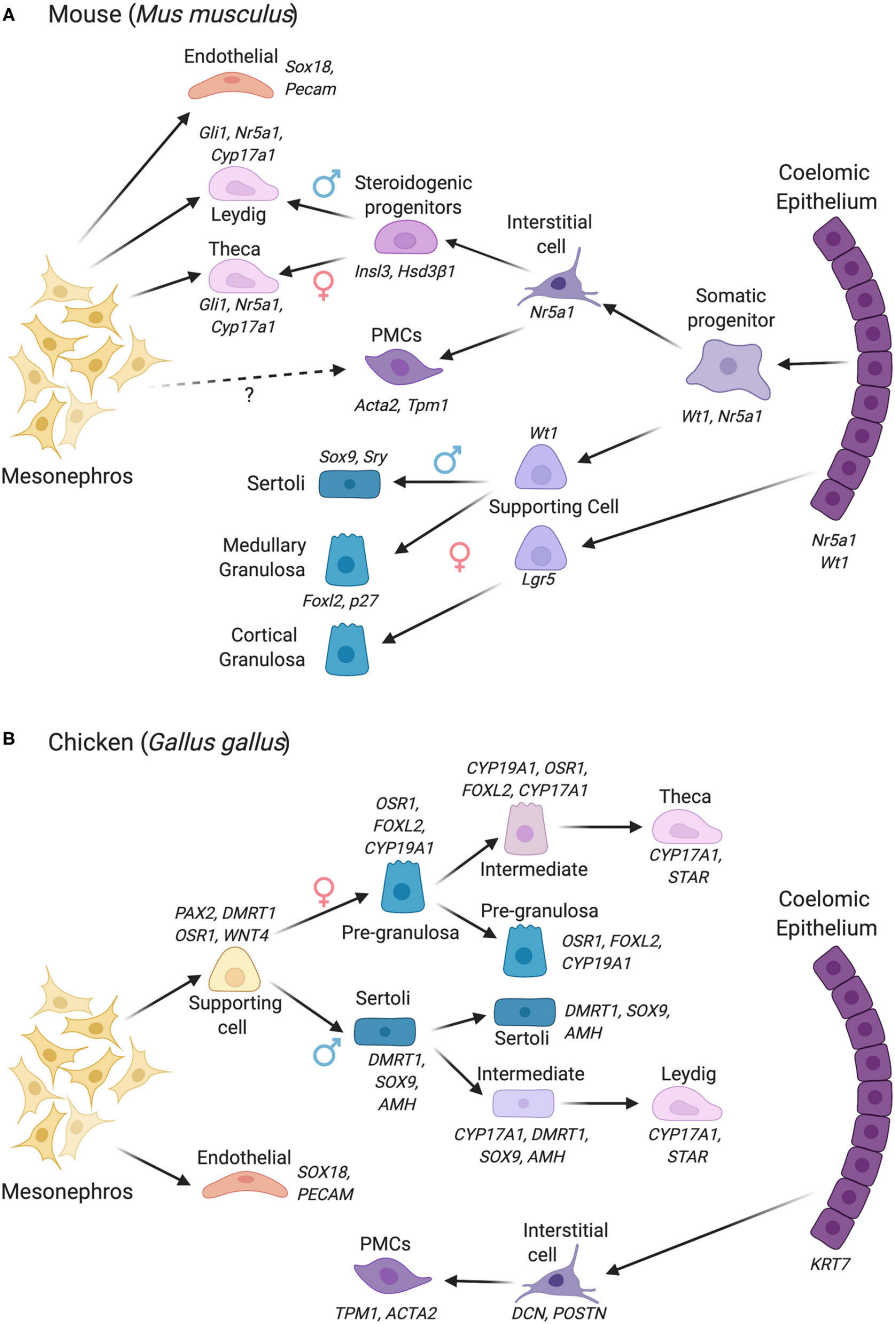
Gonadal somatic cell differentiation in the mouse vs. chicken embryo, showing major marker genes expressed. Cells derive from either the coelomic epithelium or mesonephros, with different contributions depending upon the species. **(A)** Cell lineage derivation and progression in the mouse. In mouse, cells derived from the coelomic epithelium gives rise to the supporting cell lineage and steroidogenic and non-steroidogenic interstitial cells. The mesonephric mesenchyme also contributes to the steroidogenic lineage (Leydig cells in male, thecal cells in female). In the female, two waves of supporting cell progenitors arise from the coelomic epithelium, generating medullary and cortical granulosa cells. **(B)** Cell lineage derivation and progression in the chicken, in which the supporting and steroidogenic lineages both derive from mesonephric mesenchyme. The steroidogenic lineage derives indirectly from mesonephric mesenchyme via supporting cell progenitors. This process involves the sequential upregulation of steroidogenic genes in a proportion of the supporting cells (intermediate cells), followed by a downregulation of the supporting cell markers among “intermediate” cells. The coelomic epithelium in chicken gives rise to non-steroidogenic interstitial cells. PMCs, Peritubular myoid cells. Images created using BioRender.com.

Vertebrate gonads form during embryonic or larval life and are derived from mesoderm. The gonad develops in close association with the mesonephric kidney and at the undifferentiated stage (sometimes called the “genital ridge”) it comprises cords of mesenchymal cells overlaid by coelomic epithelium (Karl and Capel, 1997; Yoshino et al., 2016; Nef et al., 2019) (Figure 1). Much of our understanding of gonadogenesis has come from studies on the mouse embryo. The somatic component of the mouse gonad largely derives from cells that proliferate from the coelomic epithelium (Karl and Capel, 1998; Schmahl et al., 2000; Schmahl and Capel, 2003; DeFalco et al., 2011). Much research has focused on the genetics and cell biology of genital ridge formation in the mouse. In this model, coelomic epithelial cells proliferate to give rise to most cells of the gonad. These cells express the gene *Nr5a1*, which encodes the transcriptional regulator, steroidogenic factor 1 (Sf1). In both mouse and chicken models, the coelomic epithelial cells undergo asymmetric cell division whereby one daughter cell remains in the epithelium and the other ingresses through an epithelial to mesenchymal transition to colonize the genital ridge (Yoshino et al., 2016; Lin et al., 2017; Yoshino and Saito, 2019). The coelomic epithelium-derived cells are considered to give rise to three main somatic cell populations in mammals: the supporting, steroidogenic and “interstitial” cells (Schmahl and Capel, 2003; Piprek et al., 2016; Stevant et al., 2018). In mouse, the first wave of cells that ingress become supporting, steroidogenic and (non-steroidogenic) interstitial cells (11.2–11.4 days post coitum, dpc). Later ingressing cells (11.5–11.7 dpc) only give rise to interstitial cells (Karl and Capel, 1998; DeFalco et al., 2011). However, it is possible that an additional source of supporting cells also exists, especially at later stages (Karl and Capel, 1998; Carre and Greenfield, 2016). In addition, gonadal somatic cells also arise from cells that immigrate from the adjacent mesonephros, giving rise to vascular endothelial cells in the testis (Brennan et al., 2002; Jeays-Ward et al., 2003; Svingen and Koopman, 2013). The mesonephros is also the source of some steroidogenic cells (Figure 2A, discussed below). Germ cells are of extra-gonadal origin; they are specified in the epiblast and migrate into the genital ridge from the hindgut and dorsal mesentery in mammals, or via the bloodstream in avians (Hen and Sela-Donenfeld, 2019).

In the early forming mouse genital ridge, cells of the coelomic epithelium express the transcription factors Gata4, Wt1, Sf1, and Lhx9, and the signaling factor Notch and its antagonist, Numb, all of which are required for proper gonad formation (Birk et al., 2000; Wilhelm and Englert, 2002; Klattig et al., 2007; Chen et al., 2017; Lin et al., 2017). Lineage tracing experiments have previously shown that a pool of Wt1^+^/Sf1^+^ multipotent progenitors cells expressing these factors in the coelomic epithelium proliferate to give rise to supporting cells and interstitial steroidogenic cells in mouse (Liu et al., 2016) (Figure 2A). A similar SF1^+^ population in bovine embryos has been termed GREL cells (Gonadal Ridge Epithelila-Like) (Hummitzsch et al., 2013). The supporting cells (presumptive Sertoli or granulosa cells) maintain Wt1 expression but down-regulate Sf1 expression, while the steroidogenic precursors maintain Sf1 and down-regulate Wt1 (Zhang et al., 2015; Chen et al., 2017). Most recently, single-cell transcriptomics has shed new light on lineage allocation in the gonads. Single-cell RNA sequencing (scRNA-seq) permits transcriptome analysis at the single cell level. Based on shared or divergent transcript profiling among cells, this approach allows the identification of novel cell type markers as well as defining the origins and developmental trajectories of cell lineages (Stevant and Nef, 2018; Estermann and Smith, 2020). In the mouse, pseudotime reconstruction based on single-cell RNA-seq data reveals that the supporting and steroidogenic progenitors indeed both derive from a coelomic epithelial population with a *Nr5a1*^+^*/Nr2f2*^+^*/Sox11*^+^ transcriptional signature. In males (XY), a subset of these cells first give rise to pre-Sertoli cells, confirming the primacy of this lineage in testis formation (Stevant et al., 2018). These cells activate *Sry* and rapidly engage a dynamic genetic program. The remaining cells subsequently give rise to waves of *Insl3*^+^*/Hsd3*β*1*^+^ steroidogenic fetal Leydig cells, and some precursors remain as non-steroidogenic “interstitial cells” (Stevant et al., 2018) (Figure 2A). The latter may contribute to adult Leydig cells, a distinct population that emerges after birth, although recent lineage tracing provides evidence that fetal Leydig cells can dedifferentiate and give rise to adult Leydig stem cells in mouse (Shima et al., 2018). In the female mouse gonad (XX), a similar pattern is followed, albeit later than in males and with less marked transcriptional changes. In females, *Foxl2*^+^ pre-granulosa cells first differentiate, followed by *Pdgfr*α^+^*/Wnt5a*^+^ cells that generate theca around birth (Stevant et al., 2019). The scRNA-seq approach outlined above has revealed a number of novel markers of cell lineage commitment in mouse gonads (Stevant and Nef, 2019; Stevant et al., 2019).

As all amniotic vertebrates exhibit the same gonadal cell types, it has long been assumed that cellular origins are conserved. However, recent scRNA-seq data from our laboratory indicate that both the supporting cell and fetal steroidogenic lineages in the chicken embryo do not derive from the coelomic epithelium (Estermann et al., 2020). This is a fundamental difference to what has been established in mouse. In chicken, the supporting and fetal steroidogenic precursors derive from a mesenchymal population that is derived from the adjacent mesonephric kidney (Figure 2B). In chicken, this population has a *PAX2*^+^*/DMRT1*^+^*/WNT4*^+^*/OSR1*^+^ molecular signature, different to that seen in mouse (Estermann et al., 2020). Gonadal *PAX2* expression appears to be unique to birds, as we did not detect the protein in embryonic mouse gonads. This finding of a non-coelomic epithelial derivation of supporting cells, discovered by scRNA-seq, supports earlier lineage tracing experiments in chicken (Sekido and Lovell-Badge, 2007). In chicken, the coelomic epithelium gives rise to non-steroidogenic interstitial cells (Estermann et al., 2020). These findings expand our understanding of gonadal cell lineage origins in vertebrates, indicating that mechanisms of assembling gonadal cell types are not conserved among clades (DeFalco and Capel, 2009). The reason for this is unclear, but additional vertebrate groups should be examined at single cell resolution to further explore the diversity of gonadal cell lineage formation.

## The Supporting Cell Lineages: Sertoli and Granulosa Cells

The supporting cell lineage is the first somatic lineage to commence differentiation in the embryonic gonad. This applies to all vertebrates that have been examined. The fate of supporting cell precursors in the gonad depends upon mutually antagonistic genetic programs (Kim and Capel, 2006; Nicol and Yao, 2015). In mouse, a critical level of *Sr*y expression during a defined developmental window activates the related *Sox9* gene, which in turn activates Fgf9, leading to pre-Sertoli cell differentiation and the formation of testis cords (Sekido and Lovell-Badge, 2008; Gonen and Lovell-Badge, 2019). In females, this pathway is antagonized by R-Spo1/Wnt4 signaling, leading to stabilization of β-catenin. In females, β-catenin signal transduction by R-Spo1 stimulates expression of Wnt4 and Follistatin, both required for pre-granulosa cell formation (Yao et al., 2004b; Chassot et al., 2008; Maatouk et al., 2008). In mouse, male-enriched Fgf9 can inhibit female-enriched Wnt4 and vice versa (Kim et al., 2006; Jameson et al., 2012a). Recent studies have shed new light on differentiation of the key supporting cell lineage. At the “bipotential” or “indifferent” stage in mouse, XX and XY gonadal transcriptomes are very similar and biased toward a female state, subsequently diverted in XY embryos by *Sry* expression (Jameson et al., 2012b). Based on scRNA-seq of sorted *Nr5a1*^+^ cells, Stévant and colleagues recently concluded that a single progenitor population in mouse gives rise to a number of somatic cell types over E10.5–E13.5; sequentially, multipotent progenitors, pre-Sertoli, Sertoli, interstitial, and fetal Leydig cells (Stevant et al., 2018). They found that Sertoli cell specification is characterized by waves of transcription factor gene expression. This includes known and new genes associated with the transient spike of *Sry* expression, several intermediate transcriptional states and a dynamic genetic program. This program features genes that repress the corresponding female developmental program and genes encoding signaling factors responsible for directing steroidogenic cell differentiation in neighboring interstitial cells, e.g., Desert Hedgehog and Pdgfα (Yao et al., 2002; Ricci et al., 2004; Stevant et al., 2018, 2019).

Single-cell RNA-seq of sorted *Nr5a1*^+^ cells and lineage tracing both indicate that the coelomic epithelium overlying the mesenchyme gives rise to the Sertoli cell population in mouse (Karl and Capel, 1998; Liu et al., 2016; Stevant et al., 2018). While it is clear that the coelomic epithelium gives rise to Sertoli cells in mouse, neither of these approaches can definitively exclude a contribution to the Sertoli cell population from mesonephric mesenchyme. Based on ultrastructural observations in human and rabbit embryos, Wartenberg and colleagues proposed a dual origin of Sertoli cells, electron dense “dark” cells emanating from the mesonephros and “light” cells derived from the coelomic epithelium (Wartenberg, 1978; Wartenberg et al., 1991). Interestingly, recent single-cell RNA sequencing in chicken suggest two potential Sertoli cell populations in E10.5 chicken testis; those expressing the typical markers, such as *AMH, SOX9*, and *DMRT1*, and those expressing these markers at a lower level and also expressing low levels of mitochondrial genes (Estermann et al., 2020). These could reflect two different Sertoli origins, but more likely reflect two different maturational stages (Guo et al., 2020). Low mitochondrial activity is linked to the stem cell state (Vannini et al., 2016), so the low mitochondrial gene expressors could be a previously unrecognized stem pre-Sertoli cell population.

In the female gonad, the supporting cell lineage differentiates into pre-granulosa cells, homologous to the pre-Sertoli cells of males (Figure 1). According to scRNA-seq, these two cell type precursors have similar transcriptional profiles, but then both up-regulate a similar set of genes, though later and temporally extended in females. Hence, commitment to a supporting cell fate is thought not to be a sexually dimorphic event (Stevant et al., 2019). In females, the presumptive pre-granulosa cells then up-regulate Wnt4 and R-Spondin1 (Rspo1), which are initially expressed in both sexes. Wnt4/Rspo1/β-catenin signaling pathway drives differentiation of the supporting cell lineage into a pre-granulosa phenotype (Kim et al., 2006; Jameson et al., 2012a; Ayers et al., 2013). Consistent with previous mouse microarray data (Jameson et al., 2012b), recent single-cell RNA-seq from both mouse and chicken indicate that the progenitor supporting cells are primed to a female (pre-granulosa) fate. In both species, undifferentiated male supporting cells cluster with differentiated pre-granulosa cells, showing strong transcriptomic similarity (Stevant et al., 2019; Estermann et al., 2020).

In the mouse ovary, pre-granulosa cells derive from the coelomic epithelium, as occurs for the male supporting cell lineage, expressing the shared signature of Sf1^+^/Wt1^+^/Gata4^+^. Two granulosa cell populations arise, which differentially express the Foxl2 transcription factor and the R-Spo1 receptor, Lgr5. The first wave of cells expresses the cell cycle inhibitor, *p27*, and *Foxl2*, forming medullary follicles. The second wave express Lgr5, then p27/Foxl2 and form both medullary and cortical primordial follicles (Mork et al., 2012; Niu and Spradling, 2020) (Figure 2A). Both granulosa cell types derive from a common Gata4 gonadal precursor in the coelomic epithelium, but the balance between Rspo1/Wnt/β-catenin and p27/Foxl2 pathways determines their differential fate (Rastetter et al., 2014; Gustin et al., 2016). In chicken, granulosa cells are characterized by maintenance of *WNT4* and transcription factor *OSR1*, and activation of the diagnostic *FOXL2* and *CYP19A1* (Aromatase) genes. In the mouse embryo, the germline plays a role in granulosa and thecal cell formation. Meiotic germ cells are required for proper differentiation of granulosa cells and folliculogenesis in the mouse embryos (Guigon and Magre, 2006). Complete thecal cell differentiation also involves inductive signaling from granulosa cells and oocytes (Liu et al., 2015). Such a critical role for germ cells does not appear to apply in the male, as testis cords can form normally without the presence of germ cells (McLaren, 1988). Using scRNA-seq, critical granulosa-oocyte interactions necessary for folliculogenesis have recently been confirmed in humans (Zhang et al., 2018). In the chicken, germ cell ablation apparently does not impact either ovary or testis formation (McCarrey and Abbott, 1978).

## Origins of the Steroidogenic Lineages

Gonads function as endocrine organs in addition to facilitating gamete production. The developmental origins of the gonadal steroidogenic cell lineages (fetal Leydig cells in males, thecal cells in females) has for some time been unclear and contested. In mouse, organ culture studies of labeled mesonephroi suggested an origin of fetal Leydig cell precursors from the mesonephric kidney (Merchant-Larios and Moreno-Mendoza, 1998; Nishino et al., 2001). Recent cell lineage tracing and scRNA-seq in mouse and chicken support a dual origin in mouse (coelomic epithelium and mesonephros) and a mesonephric mesenchymal origin in chicken (DeFalco et al., 2011; Estermann et al., 2020) (Figures 2A,B). In mouse, a second wave of coelomic epithelial cell proliferation gives rise to “interstitial” cell precursors (those outside testis cords), defined by expression of the Notch effector, *Hes1*, and others. Some steroidogenic precursors commence expression of *Gli1* in response to Dhh secreted from Sertoli cells and they form Hedgehog-dependent fetal Leydig cells (Yao et al., 2002; Liu et al., 2016). A second population of fetal Leydig cells derive from the mesonephros (Liu et al., 2016). Both populations up-regulate *Sf1* and the steroidogenic marker, *Cyp17a1*. In the early gonads of both sexes, the steroidogenic precursors are transcriptionally highly similar, essentially indistinguishable (Jameson et al., 2012b). This is because both are specialized androgen-producing cells. In females, the homologous cells are the theca, but, in the mouse, they appear much later, around birth (Nicol and Yao, 2014; Liu et al., 2015). Like the fetal Leydig cells, these have a dual coelomic epithelial and mesonephric origin in the mouse embryo (Figure 2A). Hedgehog signaling from granulosa cells induces thecal cell formation (*Gli1*^+^*/ Hsd3*β*1*^+^ cells). In addition, cells derived from the mesonephros also contribute to the thecal cell pool in mouse (Young and McNeilly, 2010; Liu et al., 2015).

In contrast to the dual origin of fetal steroidogenic cells in mouse (Figure 2A), the steroidogenic cell precursors in chicken appear to derive, like the supporting cells, directly from nephrogenic (mesonephric) mesenchyme (Estermann et al., 2020). This is another interesting difference between mouse and chicken. In fact, studies in chicken suggest that the embryonic steroidogenic lineage derives directly from the supporting cell lineage (Figure 2B). Single cell RNA-seq shows that the steroidogenic lineage progression in both sexes (fetal Leydig and thecal cells) can be traced in pseudotime to the supporting cell population (Estermann et al., 2020). A subset of supporting cells progressively up-regulate steroidogenic markers and down-regulate supporting cell markers (“intermediate” cells) (Estermann et al., 2020). This was confirmed by immunofluorescence, which shows some transitional cells expressing both supporting and steroidogenic markers during chicken gonadal sex differentiation (Estermann et al., 2020). In summary, the mesonephric kidney plays a central role in furnishing cells to the avian gonad but contributes only interstitial cell types in mouse. Further research on this point of difference could involve lineage tracing in the chicken model to confirm the mesonephric origin in chicken. This could be achieved by labeling mesonephric cells with DiI or other tracers, or generating transgenic reporter embryos with labeled mesonephric kidneys. However, at present, the production of transgenic avian embryos is time-consuming and challenging (Tyack et al., 2013; Lambeth et al., 2016).

## Other Somatic Types; The Origin and Differentiation of Non-Steroidogenic “Interstitial Cells”

In addition to supporting and steroidogenic cells, the embryonic gonad also comprises non-steroidogenic interstitial cells. These include vascular endothelial cells and peri-tubular myoid cells in males, and poorly defined interstitial cell populations in females. In mouse, there is a role for the mesonephros in the provision of interstitial cells. Male-specific migration of cells into the gonad from the mesonephros is required for proper testis cord formation in mouse (Martineau et al., 1997; Tilmann and Capel, 1999). *In vitro* gonad culture and *in vivo* reporter studies show that the immigrating cell population required for cord formation are endothelial cells, which partition cords of Sertoli cells and vascularize the developing testis (Combes et al., 2009a,b). This must involve crosstalk among mesenchymal and endothelial cells, and possibly pre-Sertoli cells. Peritubular myoid cells and other non-steroidogenic interstitial cells are thought to be induced within the mouse gonad, not migrating from the mesonephros (Cool et al., 2008). Does this apply in other vertebrates? In one study, microsurgical ablation of the mesonephros did not prevent gonadal sex differentiation in the chicken embryo (Merchant-Larios et al., 1989). However, a pool of mesenchymal cells remained after the ablation. In fact, male-specific immigration of endothelial precursor cells appears conserved in chicken (Smith et al., 2005). In chicken, at least some of these (non-endothelial) interstitial cells derive from the coelomic epithelium (Figure 2B), as shown by lineage tracing of GFP-labeled epithelial cells (Estermann et al., 2020). The fate of these cells is unclear, but they may contribute to peri-tubular myoid cells or adult Leydig progenitors. In mouse, it has been shown that macrophages derived from the yolk sac enter the male gonad are also required for proper vascular organization and cord formation in the testis (DeFalco et al., 2014). It is not known if this is a conserved phenomenon.

## Discussion and Conclusions

While the sex-determining trigger can differ among clades, vertebrate gonads typically comprise the same cell types. Recent advances in transcriptome analysis have shed new light on the cell lineage origins and developmental trajectories in the vertebrate gonad. We now have a detailed understanding of the molecular genetic programs driving supporting and steroidogenic cell lineage specification in the mouse model. The mouse may or may not be typical of all mammals. Surprisingly, the way in which these cell types are assembled differs between the mouse and chicken models. In mouse, a central role can be ascribed to the coelomic epithelium in generating the supporting cell lineage, whereas in chicken, nephrogenic mesenchyme has a central role. These apparent differences uncovered by single cell RNA-seq should now be confirmed by lineage tracing. In mouse, this can be done using inducible genetic reporters to trace lineage allocation (Zhang et al., 2015). However, transgenic technologies lag in chicken and reporter lines are not currently routine. Nevertheless, we are able to label the coelomic epithelium with GFP in chicken, which clearly shows that the epithelium does not give rise to either supporting or steroidogenic cells (Estermann et al., 2020), in sharp contrast to the mouse.

Why is there a fundamental difference in cell lineage origin between these vertebrate clades? This is unclear, but it could relate to differences in mesonephric kidney function. In mouse, there is evidence that the mesonephros is functional as an excretory organ from E10.5, before gonad formation (Lawrence et al., 2018). In chicken, the mesonephros is not fully functional until later, after the gonadal primordium is formed (Kirby, 2000). In mouse, the fact that the mesonephros is functional may impose a developmental constraint upon its involvement in gonad formation. Another possibility may involve the evolution of master sex-determining switches genes. Supporting cells in mammals are specified by *Sry*. This gene has evolved from *Sox3* and may have been co-opted to the coelomic epithelium only in the mammalian lineage. This did not occur in the avian lineage (*Sry* is absent), and hence birds retain the more ancient master sex gene, *DMRT1*, and provision of supporting cell progenitors from the mesonephros. It will be of interest to apply scRNA-seq to other vertebrate clades to test the evolutionary conservation of supporting cell lineage derivation.

The most recent embryonic gonad scRNA-seq datasets have yielded a large number of new cell type specific markers of embryonic gonadal sex differentiation (Zhang et al., 2018; Stevant et al., 2019; Estermann et al., 2020). One challenge is to now identify which of these novel factors are important for specification of the supporting and steroidogenic lineages. Gaps in our knowledge include the crosstalk between these two cell types. Signals from the supporting cells are required for steroidogenic cell specification, and the steroidogenic lineage also feeds back to the supporting cells. Further research should focus on identifying factors mediating cell cross-talk; methods such as scRNA-seq are ideally placed to address this point (e.g., see Combes et al., 2019). In chicken, a subset of supporting cells directly give rise to the steroidogenic lineages in both sexes, but how this subset is allocated is unknown. Lastly, scRNA-seq can now be combined with methods that explore gene regulation and chromatin modification at single-cell resolution, such as single-cell ATAC-seq (scATAC-seq) and ChiP-seq (Pott and Lieb, 2015; Rotem et al., 2015). This will provide a more complete understanding of the regulatory landscape governing gonadal sex differentiation. Single cell ATAC-seq identifies open chromatin associated with active regulatory regions. Integrating matched single cell RNA-seq data with scATAC-seq will allow the assembly of gene regulatory pathways activated during differentiation of the supporting and steroidogenic lineages. This information will shed light on the extent to which these pathways are shared or diverged across vertebrates.

## Author Contributions

ME and CS wrote the manuscript, while ME, AM, and CS edited the document. ME prepared the Figures. All authors contributed to the article and approved the submitted version.

## Conflict of Interest

The authors declare that the research was conducted in the absence of any commercial or financial relationships that could be construed as a potential conflict of interest.
